# CD-Based Microfluidics for Primary Care in Extreme Point-of-Care Settings

**DOI:** 10.3390/mi7020022

**Published:** 2016-01-29

**Authors:** Suzanne Smith, Dario Mager, Alexandra Perebikovsky, Ehsan Shamloo, David Kinahan, Rohit Mishra, Saraí M. Torres Delgado, Horacio Kido, Satadal Saha, Jens Ducrée, Marc Madou, Kevin Land, Jan G. Korvink

**Affiliations:** 1 Council for Scientific and Industrial Research, Meiring Naude Road, Brummeria, Pretoria 0001, South Africa; kland@csir.co.za; 2 Institute of Microstructure Technology, Karlsruhe Institute of Technology, Hermann-von-Helmholtz-Platz 1, Eggenstein-Leopoldshafen 76344, Germany; dario.mager@kit.edu (D.M.); jan.korvink@kit.edu (J.G.K.); 3 School of Engineering and School of Physical Sciences, University of California, Irvine, 4200 Engineering Gateway, Irvine, CA 92697-3975, USA; aperebik@gmail.com (A.P.); eshamloo@uci.edu (E.S.); hkido@uci.edu (H.K.); mmadou@uci.edu (M.M.); 4 School of Physical Sciences, Dublin City University, Glasnevin, Dublin 9, Ireland; david.kinahan@dcu.ie (D.K.); rohit.mishra@dcu.ie (R.M.); jens.ducree@dcu.ie (J.D.); 5 Simulation Laboratory, Department of Microsystems Engineering (IMTEK), University of Freiburg, Freiburg im Breisgau 79085, Germany; sarai.torres@imtek.de; 6 Foundation for Innovations in Health and JSV Innovations Private Limited, 44A S P Mukherjee Road, Kolkata 700026, India; drsatadal.saha@gmail.com

**Keywords:** primary care, extreme point-of-care, centrifugal microfluidics, CD-based microfluidics, lab-on-a-disc

## Abstract

We review the utility of centrifugal microfluidic technologies applied to point-of-care diagnosis in extremely under-resourced environments. The various challenges faced in these settings are showcased, using areas in India and Africa as examples. Measures for the ability of integrated devices to effectively address point-of-care challenges are highlighted, and centrifugal, often termed CD-based microfluidic technologies, technologies are presented as a promising platform to address these challenges. We describe the advantages of centrifugal liquid handling, as well as the ability of a standard CD player to perform a number of common laboratory tests, fulfilling the role of an integrated lab-on-a-CD. Innovative centrifugal approaches for point-of-care in extremely resource-poor settings are highlighted, including sensing and detection strategies, smart power sources and biomimetic inspiration for environmental control. The evolution of centrifugal microfluidics, along with examples of commercial and advanced prototype centrifugal microfluidic systems, is presented, illustrating the success of deployment at the point-of-care. A close fit of emerging centrifugal systems to address a critical panel of tests for under-resourced clinic settings, formulated by medical experts, is demonstrated. This emphasizes the potential of centrifugal microfluidic technologies to be applied effectively to extremely challenging point-of-care scenarios and in playing a role in improving primary care in resource-limited settings across the developing world.

## 1. The Need for Extreme Point-of-Care

In resource-limited settings, for example in parts of India and Africa, access to everyday commodities, such as clean water and electricity, is restricted. This makes day-to-day living in the developing world vastly different from the experiences of first world settings and even more so when it comes to healthcare. In these low-infrastructure settings, exposure to difficult environmental conditions is commonplace, including high levels of humidity, heat and dust. Electricity is often intermittent or non-existent, compounding the harshness of the environment and posing significant challenges for equipment and data connectivity. In addition, lack of trained staff makes it hard to provide a high standard of diagnostic testing and throughput of patients. Clinics are remotely situated, and patients need to travel, often over great distances, to seek medical assistance. Samples collected at the clinics need to be sent to a centralized laboratory with a waiting period to receive test results, and frequently, patients fail to return to the clinic for the diagnoses as a result of travel time, prohibitive costs and possibly even social pressure.

In addition to the lack of doctors, nurses and hospital beds, primary care is insufficient for those living in third world settings in rural parts of India and Africa. In India, for example, approximately 30% of the population does not have access to primary care [1]. This lack of primary care, coupled with the absence of health education, leads to the accumulation of disease burden in society, where patients often present in advanced disease states, requiring expensive secondary and tertiary care. A total of 39 million people fall below the poverty line in India every year alone as a result of health-related expenses [1]. Human and insect communicable diseases, such as tuberculosis (TB), malaria and hepatitis, are prominent in under-resourced settings [2], and timely diagnoses of these conditions are urgently needed. Providing comprehensive primary care in under-resourced settings is a paramount global challenge, which can be clearly addressed by innovative, effective point-of-care (POC) diagnostic technologies, which are compatible with these extreme environments.

Significant humanitarian, social and economic benefits can be derived from such POC technology initiatives. Delivery of primary care in extreme POC conditions close to peoples’ homes makes it possible for women and children to benefit from care; and this has important implications. For example, it is estimated that 20%–40% of maternal deaths in India result from anemia, with the prevalence of anemia among pregnant women in India upwards of 40% and India contributing to approximately 50% of global maternal deaths due to anemia [3]. This can be corrected through iron-folic acid supplementation through a primary care center. Iron deficiency anemia is estimated to cause 591,000 perinatal deaths globally. The associated loss of healthy life years amounts to more than 19 million disability-adjusted life years (DALYs) from perinatal causes. It has been concluded from large meta-analytical studies that there is significant reduction in perinatal risk, concurrent with maternal iron-folic acid supplementation [4]. Providing effective healthcare forms part of global millennium development goals [5], as well the millennium development goals for India [6] and the National Development Plans for South Africa [7], and examples such as those discussed above emphasize the importance of combining socio-economics with technology to ensure that primary care is comprehensive in resource-poor settings.

Advances in the development of POC diagnostics have accelerated in recent years [8], with initial success seen in first world settings, such as hospitals and doctor’s offices, where skills and the environment pose fewer constraints than in the developing world. Emphasis is now shifting towards the development of POC solutions for the developing world [9], also known as extreme POC solutions, to address the striking demand for effective healthcare where the need and the impact is highest. UNITAID , a global health initiative that focuses on addressing diseases, such as human immunodeficiency virus (HIV) and acquired immunodeficiency syndrome (AIDS), tuberculosis and malaria in developing countries, produces annual reports on diagnostic developments for many of the main diseases prevalent in developing countries, such as HIV [10], malaria [11], hepatitis [12] and tuberculosis (TB) [13].

Extreme POC tests are required to address many limitations, including power, operators’ skills and environmental conditions. New technologies also need to be developed for POC tests, as conventional lab-based diagnostic technologies are often too expensive and complex to operate and would not be feasible options at the POC. This article highlights the need for POC systems along with developmental pathways for these emerging technologies.

The World Health Organization (WHO) has set out the ASSURED criteria (affordable, sensitive, specific, user-friendly, rapid and robust, equipment-free and deliverable to end users), the minimum requirements to which POC diagnostics should conform [14]. The ASSURED criteria are particularly relevant to extreme POC settings and have been examined with specific application to the developing world and under-resourced areas [2,9]. The criteria have also been investigated for existing important tests, such as CD4 counts for monitoring of HIV [15], where commercial systems are evaluated according to their fit to the ASSURED principles. Glynn *et al.* also noted that an important additional requirement for emerging POC technologies is their compatibility with concurrently emerging trends and technologies [15]. The ASSURED principles provide an ideal to work towards, but practical limitations still exist, for example where eventually equipment-free solutions may require minimal instrumentation as a first step.

Even with the definition of the ASSURED criteria and the well-known need for POC solutions for under-resourced environments, particularly in parts of India and Africa, little commercial implementation has been achieved. WHO and the Gates Foundation, as well as other organizations, have established various funding streams supporting research towards effective POC diagnostics for under-resourced settings. Programs, such as Beyond Traditional Borders (BTB) [16], have resulted in innovations, such as the lab in a backpack, consisting of compact equipment, including an oil immersion microscope and a battery pack to last for up to eight hours, for distribution in rural clinics.

In resource-limited settings in South Africa, for example, a number of clinics have been set up, and training has been provided in an attempt to improve healthcare in under-resourced settings [17]. However, numerous challenges remain within the testing environments themselves in South Africa and include specimen collection methods, lack of skilled staff and lack of or inconsistent quality assurance practices [18]. Sample collection poses a major roadblock in current POC tests. Small sample volumes are used, and small variations in the volume typically result in errors. POC tests are also often stigmatized by healthcare workers as time consuming and complicated to handle, thus significantly increasing their workload [19]. These issues illustrate the importance of developing accurate POC tests that are fully sample-to-answer automated, with little user interaction or skill required, to overcome these barriers to widespread acceptance of POC solutions for under-resourced settings. Adaptability to existing training, workflow and environmental restrictions in under-resourced settings needs to be taken into account for POC diagnostic systems in order to become an integrated part of technology-enabled primary care solutions [20].

**Figure 1:**
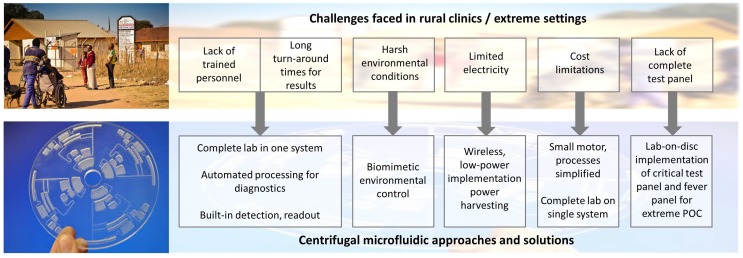
Overview of topics discussed in this paper, including the challenges faced in rural clinics and corresponding CD-based technologies for addressing these issues.

Figure 1 summarizes the various challenges faced in rural clinics in under-resourced settings, with corresponding centrifugal microfluidic technologies and developments. Limitations, such as access to trained staff and laboratory equipment, in extreme settings can be potentially overcome by utilizing an automated, complete lab-on-a-disc centrifugal system, as discussed in Section 4.1. Challenges such as electricity limitations and extreme environmental conditions, such as high temperature, humidity and dust levels, can be targeted through low-power centrifugal system implementations and biomimetic approaches for energy harvesting and environmental control, as outlined in Section 4.2.2 and Section 4.2.3, respectively. The simplicity of implementing centrifugal systems while achieving advanced and diverse functionality enables issues, such as cost and complete test panels, to be addressed. This paper discusses the various challenges faced in extreme POC settings and the potential CD-based microfluidic advances that could assist in formulating solutions.

## 2. Fluidic Approaches

The development of technical components for POC systems requires sample and reagent handling, manipulation and analysis. The field of microfluidics encompasses the precise and automated control of very small volumes of fluids, usually on a nanoliter scale. Microfluidic systems are often referred to as lab-on-a-chip systems or micro-total-analysis-systems (MicroTAS). Microfluidic technologies utilize small liquid volumes in compact, disposable devices, enabling rapid reaction times, automated fluidic handling and cost-effective use of materials and reagents. These advantages make microfluidic systems well-suited to a broad range of predominantly (bio-)analytical applications, including POC diagnostics. A sizeable spectrum of microfluidic platforms have been developed in the recent past, which are typically distinguished by their actuation principle, including: pressure, capillary action, electrokinetics, acoustics and centrifugation; a number of excellent reviews showcase these technologies and their applications [21,22,23]. The use of microfluidic systems specifically for the development of POC diagnostics has also been explored [24,25,26,27,28,29].

The emerging field of paper-based microfluidics combined with smart phone-based technologies shows promise for extreme POC diagnostics. Paper is low-cost and disposable, and smart phones are widely accessible, providing a powerful platform for POC diagnostics. This work specifically addresses centrifugal microfluidic systems as applied to extreme POC and does not include reviews of other microfluidic technologies suited to extreme POC. For further insight into such technologies, the reader is referred to a number of excellent reviews and recent work in the field of paper-based and smart phone-based diagnostic technologies [30,31,32,33,34].

Centrifugal microfluidic systems, also referred to as lab-on-a-disc or lab-on-a-CD solutions, provide a particularly attractive solution for the implementation of microfluidic POC diagnostic systems [35,36]. Centrifugal microfluidic technology makes use of a disc, similar in size and shape to a CD or DVD, to house microfluidic channels and features. A motor is used to rotate the microfluidic disc, transporting fluid radially outwards through the microfluidic device, and manipulating fluid by means of various microfluidic functions and features on the disc.

In addition to the general advantages of microfluidic systems regarding POC applications, including small sample and reagent volumes, tight control of fluidic functionality, short diffusion distances and compact, disposable devices, centrifugal systems provide some further benefits [37]. These include the simple and compact external instrumentation required: only a small rotating motor is required to achieve a vast assortment of complex fluidic functionality, in contrast to bulky, expensive pumps or high voltages that are often required to drive fluids in other microfluidic technologies [38]. The disc format of centrifugal microfluidic devices lends itself to effective multiplexing of tests on one device as a result of rotational symmetry, which also enables a high throughput of tests [39]. Simple actuation principles are used for centrifugal technologies, and thus, clean, modular separation between the disposable disc and the drive or readout unit can be achieved [40].

As a result of these foundational advantages, as well as further advances and innovations that will be discussed in this paper, we believe that CD-based microfluidic technologies have the potential to provide extreme POC solutions towards effective primary care in under-resourced settings.

## 3. Introduction to CD-Based Microfluidics

### 3.1. Theory of Operation

**Figure 2:**
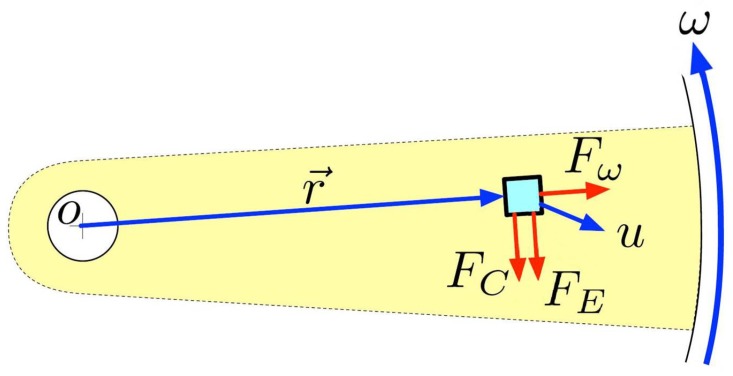
Illustration of forces utilized to control fluids on centrifugal microfluidic platforms, including the centrifugal force (*F*${}_{\omega }$), Coriolis force (*F*${}_{C}$) and Euler force (*F*${}_{E}$). Kinematic quantities (blue) and forces (red) are shown for a fluid element moving at a velocity (*u*) on a portion of a disc at a distance $\bar{r}$ from the center of the disc.

Centrifugal microfluidics, or lab-on-a-disc systems, make use of three pseudo forces present on a rotating platform, *i.e.*, centrifugal, Coriolis and Euler forces, to effectively propel and control fluids within disc-shaped devices. These forces are illustrated in Figure 2.

The centrifugal force, acting radially outward and proportional to the square of the angular velocity, is the primary force used to move fluid from the center to the edge of the disc; flow rate depends on fluidic properties, such as density and viscosity, the angular velocity of the disc, channel geometry and the radial location of fluid on the disc. The average velocity of liquid on a disc is given by Equation (1) as:

(1) $$\vec{U}=\frac{D_{b}^{2}\rho {\vec{\omega }}^{2}\bar{r}\Delta r}{32\mu L}$$

where $D_{b}$ is the hydraulic diameter of the channel, *ρ* is the liquid density, $\vec{\omega }$ is the angular velocity, $\bar{r}$ is the average distance of the liquid from the center of the disc, Δr is the radial extent of the liquid, *μ* is the viscosity of the liquid and *L* is the length of the liquid column in a channel or chamber on the disc.

This centrifugal pumping mechanism has been used to successfully pump a variety of liquids in lab-on-a-disc systems, widely independent of their physico-chemical properties, demonstrating its effectiveness in biological applications where it is important to be able to handle a large range of liquid types and volumes on the same disc [41].

The Coriolis force, which is perpendicular to the velocity of a moving particle on the disc and directly proportional to both the mass and the cube of the spin speed of the disc, is frequently used for switching the direction of flow [42], as well as for density-based particle separation and sorting on the disc [43,44]. Particles are sent along different path trajectories through chambers on the disc based on their differing masses allowing for effective separation of key biological components [45].

The Euler force emerges opposite to the rotational acceleration in the plane of the disc. In lab-on-disc systems, the primary function of the Euler force is in mixing to create lateral motion of the fluid during disc acceleration.

The combined pseudo forces, per unit volume, on a particle or liquid droplet on a disc, are shown in Equation (2) as:

(2) $${\vec{F}}_{\text{tot}}=\rho \vec{\omega }\times \left(\vec{\omega }\times \vec{r}\right)-2\rho \vec{\omega }\times \frac{d\vec{r}}{dt}-\rho \frac{d\vec{\omega }}{dt}\times \vec{r}$$

where *ρ* is the liquid density, $\vec{\omega }$ is the angular velocity in rad/s, $d\vec{r}/dt$ is the velocity vector of the particle moving on the disc, $d\vec{\omega }/dt$ is the angular acceleration and $\vec{r}$ is the average distance of the liquid from the center of the disc. The terms represent the centrifugal force, the Coriolis force and the Euler force, respectively.

The use of these pseudo forces affords a wide range of control to the user in liquid manipulation with minimal outside hardware, making centrifugal microfluidic systems well suited to POC diagnostic applications [35,46]. Centrifugal microfluidic systems thus bear the potential to distinctively address many challenges associated with delivering POC diagnostics to extreme settings.

### 3.2. Recent Advances in CD Fluidics

Centrifugal or CD-based microfluidics allow for complex liquid handling to be implemented using simple mechanisms through the theory presented in Section 3.1. Advances in CD fluidic technologies provide the ability to effectively implement sophisticated mixing and valving operations, as well as cell handling.

#### 3.2.1. Mixing

**Figure 3:**
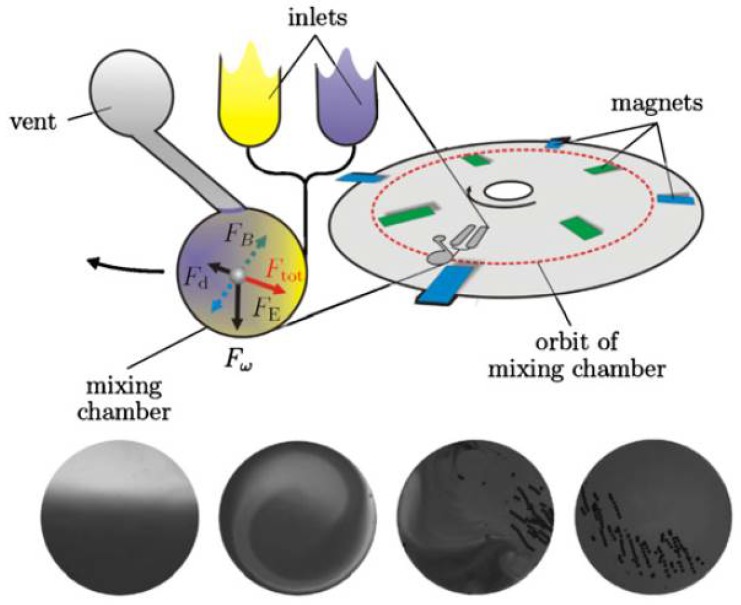
Illustration of rapid mixing techniques in centrifugal microfluidic systems based on the work by Grumman *et al.* [47], where magnets are used to facilitate mixing. Permanent magnets are aligned on the set-up at radial positions that are inbound and outbound relative to the mean orbit (dashed circle) of the rotating mixing chamber. A magnetic bead inside the mixing chamber is thus exposed to an alternating radial force *F*${}_{B}$ and a viscous drag force *F*${}_{d}$, in addition to the centrifugal force *F*${}_{\omega }$ and Euler force *F*${}_{E}$. The bottom row of images shows the flow patterns within the mixing chamber. Image reproduced from [46]. © IOP Publishing. Reproduced with permission. All rights reserved.

Mixing, which is aggravated by the laminar conditions prevalent in microstructures, can be enhanced in centrifugal microfluidic systems using inherent forces created by spinning the disc. Entrapment of air bubbles is also less problematic than in many other microfluidic systems, as they are eliminated in the buoyancy induced by the centrifugal field, driving the gas centripetally towards the top, *i.e.*, radially the inner side of the microfluidic chamber. Grumann *et al.* have demonstrated mixing of liquids with beads using two different approaches. In the so-called “shake mode”, the mixing is achieved using inertia effects to induce stirring of the liquid based on the change in the disc spin direction [47]. The other method demonstrated was specifically applicable to mixing of magnetic beads, where magnets set in a fixed frame on the disc were used to periodically deflect the particles due to interaction of the beads with a static, external magnetic field. A combination of the two techniques demonstrated mixing of beads with liquids within a second (Figure 3). Ducrée *et al.* applied convective mixing that is dictated by velocity-dependent Coriolis pseudo force and the interaction of the transverse currents with the side walls [48]. The key parameters that influenced this advection under laminar conditions were the geometry and the speed of rotation for shortening mixing times.

Noroozi *et al.* have demonstrated mixing of nano- and micro-liter volumes using a specifically-designed device that induced localized chaotic flow using a combination of rotationally-induced forces, reciprocating flow and oscillatory volume contractions [49]. The micromixer unit consists of two reservoirs (initial storage of the two fluids to be mixed), a pneumatic pressure chamber and a mixing chamber connected by microchannels fabricated in polydimethylsiloxane (PDMS). The reciprocating flow induced by the centrifugal force and the resulting pneumatic pressures enhanced the mixing for small volumes of up to 30 μL. Recently, Clime *et al.* have demonstrated an active mixing platform that is implemented using system integration of a pressure pump and a programmable electromechanical valving scheme [50]. A fast pressure pulse using the pumping enabled by the control of pneumatic pressure in the connected microfluidic network allows for ultra-fast bubble production through a gas inlet at the bottom of the mixing chamber. The centripetal acceleration and the buoyancy forces generated are significant in the rotating platform by allowing the gas to escape from a top vent channel before the formation of larger bubbles that can cause significant liquid flow out of the chamber. Robust, high performance liquid handling and the use of unique artificial gravity conditions hence enable mixing on a centrifugal platform using passive and active techniques.

Mixing is a crucial element for the integration of full bioanalytical process chains, from sample preparation through to assay implementation and detection, and centrifugal microfluidic systems have the capability to implement this functionality effectively.

#### 3.2.2. Valving and Timing Control

**Figure 4:**
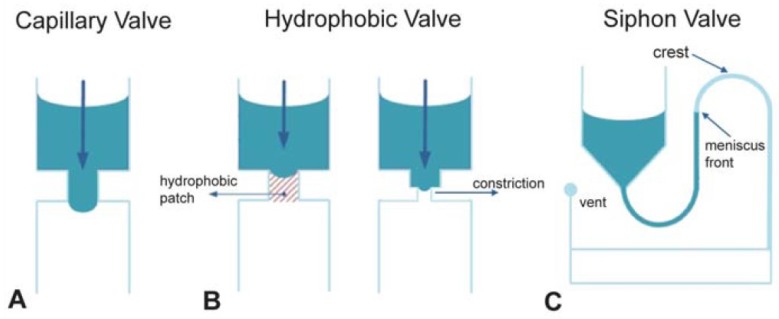
Examples of commonly-used valving techniques in centrifugal microfluidic systems. (**A**) Capillary valving implemented by a sudden expansion in the channel diameter; (**B**) hydrophobic valving using either a hydrophobic patch made by applying hydrophobic material to a zone inside a channel or by implementing a constriction in a chamber or channel made of hydrophobic material; (**C**) siphon valving implemented using a reservoir and an outlet connected by a hydrophilic channel. At high spin speeds, centrifugal forces keep the meniscus front below the crest. When the spin speed is reduced to below a critical value, the channel is primed, and the liquid is transferred as soon as the meniscus passes the crest point. Reproduced in part from [35] with permission of The Royal Society of Chemistry.

Valving for flow control on disc is particularly critical as a result of the unidirectional nature of the centrifugal force. Commonly-used centrifugal microfluidic-based valving methods include capillary, hydrophobic and siphon valves, as shown in Figure 4. Broadly, most valving techniques can be divided into rotationally-controlled and instrument-supported valves that can be actuated (widely) independently of the speed of the system-innate spindle motor. The latter, externally-actuated centrifugal platforms can be categorized by their interaction mechanism with the disc. This can include external pressure sources [51] for on-disc flow control, heating to induce phase changes in material, such as ferrowax [52,53,54,55], or even physical manipulation [50,56]. Instrument-supported valves typically provide enhanced process control, however, typically at the expense of requiring additional instrumentation. Yet, the shrinking cost and ubiquity of microcontrollers increases the feasibility of instrument-supported platforms for POC in extreme environments. This includes pneumatic pumping and controlled reorientation of the chip during rotation [57]. Furthermore, mechanisms for wireless energy transfer to a rotating disc [58], as discussed in Section 4.2.2, have the potential to greatly enhance the capabilities of the centrifugal platform.

**Figure 5:**
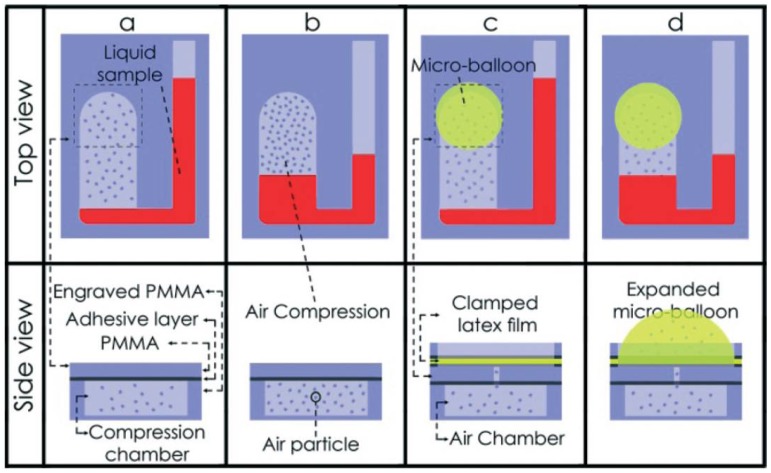
Example of using micro-balloons as flexible walls for effective pneumatic siphon valving in centrifugal microfluidics using lower spin speeds. A comparison is given between standard pneumatic pumping (**a**,**b**) and pneumatic pumping with the micro-balloon (**c**,**d**). The micro-balloon implementation (c,d) requires a much lower angular velocity than the standard implementation (a,b), which has a higher air pressure at high centrifugal forces. Reproduced from [68] with permission of The Royal Society of Chemistry.

The former, rotationally-actuated valves typically depend on the interplay of surface forces (such as interfacial tension) with the body forces (centrifugal force) acting on the liquid co-rotating with the disc. Changing the centrifugal force is often used for destabilizing the equilibrium of forces, e.g., for opening a valve. Due to their dependence on the contact angle, these valves usually depend quite sensitively on surface finish and manufacturing tolerances of the disc. These rotationally-actuated valves include capillary valves [59,60], dead-end pneumatic chambers [61], dissolvable film (DF) valves [62], burstable foils [63] and elastomeric membranes [64]. Triggered by a reduction in spin rate, low-pass valves are typically based on siphon structures [65]. This basic structure is often enhanced through pneumatic pockets [66,67], where pneumatic pressure is stored during disc acceleration and released at low spin rates, e.g., to prime siphons, which are even slightly hydrophobic. The performance of such siphons can also be varied through using pneumatic pockets with flexible walls [68], as illustrated in Figure 5.

The number of rotationally-actuated valves that can be integrated into a system is naturally limited by the practical number of burst frequency bands available. In practice, the upper frequency at which a disc will function is defined by the minimum achievable feature size. In addition, manufacturing tolerances smear the geometrically-defined burst frequencies of these valves into bands [69]. This limitation has been circumvented in a number of ways. For example, Siegrist *et al.* successfully combined low-pass siphon valves placed in series with high-pass capillary valves, which open using a downwards pulse in the spin frequency [65]. Schwemmer *et al.* used liquid resistance to stagger, in time, the actuation of pneumatic siphons [70]. Others have made efforts to develop valving mechanisms, which are independent of the spin rate. Often, these triggering mechanisms are based on liquid movement about the disc [71]. Kinahan *et al.* introduced so-called “event-triggered valves”, which function upon this principle of liquid movement [69]. These DF-based valves function in a manner akin to a single-use electrical relay; through a circuit of pneumatic channels and the dissolution of a film, the arrival of liquid at one point on the disc triggers the release of liquid at a distant location. These circuits can also be combined to control valve actuation based on flow that meets logical conditions, such as AND and OR. A limitation of this platform is the timing of valve actuation, which translates to the time for DFs to dissolve and liquid to move about the disc. In order to offer enhanced process control, paper strips have been integrated on-disc to time liquid handling on the disc [72].

Recently, Mishra *et al.* demonstrated flow control and routing using a multi-material approach where DFs only dissolve in the presence of specific liquids [73]. The DF membranes are fabricated from specific materials that are both compatible and immiscible in aqueous solutions that are common in diagnostic assays [74]. They enable a barrier formation between channels that require separation; for instance, in the case of a router where an alternative channel needs to be opened to allow flow from one line to the other in a multi-step process. The centrifugal field enables the stratification of the immiscible solutions, thus allowing the distinct separation of the aqueous phase from the oil phase, as well as triggering DF valves fabricated from different materials on demand. The approach provides a low-cost and instrumentation-free alternative to active routing strategies, as it is entirely triggered by rotation.

**Figure 6:**
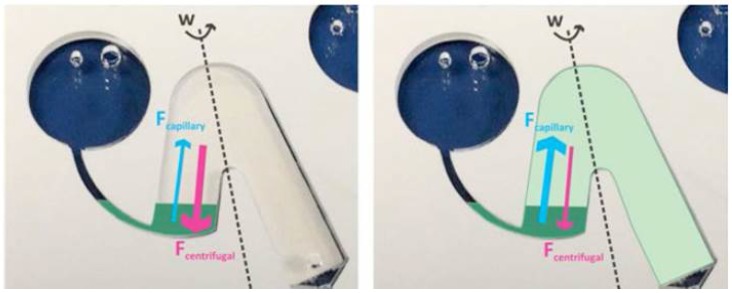
Example of hybrid paper-on-a-disc device using a paper siphon in a chamber on a microfluidic disc. Opposing effects of centrifugal and capillary forces are shown for $F_{centrifugal}$ > $F_{capillary}$ (**left**) and $F_{capillary}$ > $F_{centrifugal}$, where liquid is absorbed by the paper (**right**). Reproduced from [81] with permission from the Chemical and Biological Microsystems Society, CMBS.

Unidirectional flow in centrifugal microfluidics is perceived as a limitation of these systems. Particularly, the need to store reagents at the center of the disc, where real estate is both most limited and most valuable, has driven the developments of centripetal pumping methods. These include thermo-pneumatic pumping [75], micro-pumps integrated into the spindle motor [50], electric power driving on-disc electrolysis [76] and chemical pumping [77]. Rapid deceleration of the disc coupled with pneumatic pockets has also been utilized for centripetal pumping [70,78]. Furthermore, pumping based on positive and negative displacement using secondary liquids has been demonstrated to good effect [51,79,80]. Additionally, the incorporation of paper on disc devices [81,82,83,84] also permits centripetal pumping. These hybrid paper-on-a-disc solutions also combine the increased capillarity of paper with the changeability of the counteracting centrifugal forces to provide additional fluidic handling capabilities. An example of a hybrid paper-on-a-disc device is shown in Figure 6.

#### 3.2.3. Cell Handling

Their intrinsic centrifugation capability makes the spinning disc platform particularly advantageous for applications where density-based separation of bioparticles, such as cells and beads, are required [40,43]. Large volume separation of plasma from whole blood was demonstrated by Amasia *et al.*, 2010 [85], while more recently, the capture of bacteria in V-shaped structures to detect urinary tract infections through optical counting of bacteria in a urine sample has been demonstrated [86]. Similarly, isolation of white blood cells from whole blood using density gradient media (DGM) has also been shown [87,88,89]. Furthermore, DGM centrifugation has been used for diagnosis of eye disease [90] and the detection of toxins [91].

Rare cell detection in patient samples, typically blood, is of major diagnostic value; the detection of circulating tumor cells on centrifugal microfluidic platforms has been implemented, driven by inertia, magnetophoretic separation and capture of micron-scale obstacles. Kirby *et al.* have introduced centrifugo-magnetophoretic particle separation that utilizes the effects of the magnetic deflection of particles sedimenting in stopped-flow mode under the impact of the centrifugal field. Paramagnetic beads that specifically bind to target cells in whole blood are separated from background cells and unbound beads by the interplay of the centrifugal force, lateral magnetic force on the disc and the hydrodynamic Stokes drag [92]. Glynn *et al.* have demonstrated the size separation of clustered cancer cells using a microstructure rail embedded in a disc cartridge [93]. The rail shows a series of openings with increasing aperture width that allow only clusters below a certain size to pass while diverting larger particles. Burger *et al.* developed a centrifugal platform for the capture and optical detection of cancer cells and beads using microstructure-based geometrical trapping. An array of scale-matched microstructures along the centrifugal axis efficiently traps the cells and also allows for staining and optical analysis for further characterization. An optical trap-based laser tweezer was also integrated into the system for transferring single cells from the traps to designated locations for further investigation [94].

Lee *et al.* have also demonstrated the separation of circulating tumor cells from whole blood on a plastic centrifugal disc platform with a polycarbonate filtration membrane that is embedded between a sample chamber and a waste chamber. The platform allows the cells to be sorted from the smaller red and white blood cells within 20 s while handling a larger volume of whole blood, instead of a few hours, as in the case of immunoaffinity-based isolation platforms [95]. Park *et al.* have demonstrated circulating tumor cell (CTC) isolation from whole blood on a fully-automated centrifugal disc that incorporates ferrowax valving technology in combination with a DGM. Microbeads that are functionalized with specific antibodies to CTCs are mixed with whole blood after plasma removal in the disc. This leads to a clear density difference between tagged CTCs and blood cells and then allows CTCs that are now heavier than blood cells to settle under centrifugal force in a DGM [96]. Aguirre *et al.* have demonstrated a combined cell-bead micromixer unit and an inertial flow separation and detection structure on a centrifugal platform. Dean flows in curved channels enhance the mixing of functionalized beads to CTCs, while a combined transversal movement of particles in channels with constant centrifugal force aligned the bead-tagged cells along the wall, eventually leading to their separation [97].

**Figure 7:**
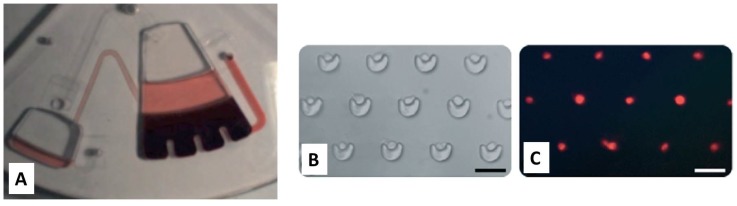
(**A**) Blood plasma separation on a microfluidic disc from a large sample (>2 mL) of blood. Image reproduced from [85] with permission from Bioanalysis as agreed by Future Science Ltd. (**B**) Brightfield microscope image of individual HL60 cells captured in V-cup arrays; and (**C**) fluorescence imaging of the labeled cells. Scale bar = 50 μm. Reproduced in part from [94] with permission of The Royal Society of Chemistry.

Some examples of advances in cell handling in CD-based microfluidics are shown in Figure 7. A number of excellent reviews on centrifugal microfluidic or lab-on-a-disc technologies provide insight into the mechanisms utilized in CD-based fluidics and highlight the functional building blocks, integration and advantages of centrifugal microfluidics [37,98].

## 4. Centrifugal-Based Systems for Extreme Point-of-Care

More recently, there has been a strong drive towards the implementation of microfluidic systems for POC diagnostics in resource-limited or extreme settings [99,100,101], but many challenges remain in producing viable commercial devices. A number of reviews highlighting their applicability to POC and biomedical applications [35,46,102] demonstrate the potential of CD-based microfluidics to realize extreme POC solutions. Some of the key advantages of centrifugal-based systems to achieve this are discussed in the sections that follow.

### 4.1. Lab on a CD

In addition to their functional blocks that are advantageous over existing microfluidic techniques for POC applications, CD-based systems can emulate existing standard laboratory equipment. CDs are traditionally played using a Discman® (Sony Corporation, Tokyo, Japan) or a CD-ROM, and this surrounding CD infrastructure, including a servo-motor for spinning the disc, a laser and lens system on the “pick-up” head for optical detection and a tracking system, which moves the laser to different radial distances along the disc, can be viewed as an all-in-one lab: An integrated system that can perform a number of standard laboratory procedures. In addition, CD-based microfluidics utilize low-cost consumables, commonly in the form of polycarbonate disposable discs, which are well-suited for performing various lab tests. Different laboratory equipment functions that can be realized by centrifugal microfluidics and their surrounding infrastructure are discussed in the following paragraphs and are summarized in Table 1.

**Table 1:** The utility of a CD player as a diagnostic instrument with example applications.

Laboratory EquipmentFunction	Implementation Method	Example Applications
Centrifuge	Motor for spinning disc. High speed for centrifuge action. Motor speed can be varied accurately.	Blood plasmaseparation [85].
Vortex	Motor for spinning disc clockwise or counter-clockwise. Turbulence created causing vortex effect. Rotation direction changes vary acceleration, causing turbulence.	RotaPrep, Inc. [103], cell culture [104], vortexing [47].
Mixer	Ceramic beads incorporated into disc. Mixing of fluids using beads when turbulence created. Changes in spin direction cause turbulence and mixing.	Mixing of different reagents [47].
Lysis	Magnets and glass beads incorporated into disc. Lysis of cells as a result of mechanical impaction and shear forces. Rotational dual magnetic field moves magnet and beads inside chambers.	Lysis of bacteria [105,106], RotaPrep, Inc. [103].
Microscope	CD or DVD player. System components used as laser scanning microscope. Photodetector module detects absorbance of objects, images reconstructed.	Detection of cells [107,108].
*x*-*y* table/spotter	CD or DVD player. Rotational and linear motors for positioning. Microarrays applied onto disc using piezoelectric inkjet applicator and positioning system.	Immunoassay microarrays [109].
Sample concentration	Pneumatic pressure chambers on a disc. Reciprocation pump implemented. Fluid flushed back and forth, concentration/capture of analyte.	Immunoassays [49].
Cell counter	CD or DVD player. Locating and counting of cells, microparticles, biomolecules. Laser in drive detects errors on disc where particles are located.	Counting of cells, microparticles [107,110].
Thermal cycler	Peltier elements. Thermal treatment of small chambers. Current direction changes mode from heating to cooling.	DNA amplification via PCR [111].
Hypergravity simulation	Spinning microfluidic disc. Chambers containnutrients for cultivation. Centrifugal force simulateshigh *g*-forces on live samples trapped in fluidic chambers.	*C. elegans* stress response cultivation platform [112].

The primary equipment that a microfluidic disc emulates is that of a standard laboratory centrifuge. The servo-motor that propels liquid can also be used for density-based separation (in stopped-flow mode). Amasia *et al.* [85] used a microfluidic disc to demonstrate rapid and automated separation of red blood cells from plasma, a task traditionally performed by lab centrifuges. The disc also makes a functional laboratory vortex or mixer through the use of the pseudo forces introduced through rotation of the disc. In shake mode, the angular acceleration of the disc induces chaotic advection to rapidly vortex a sample [47]. By placing a series of permanent magnets below the disc and both magnetic and small glass beads inside the disc chamber, the shear forces are sufficient to rupture cell walls in biological samples, allowing the disc to also function as a mechanical lyser [105].

**Figure 8:**
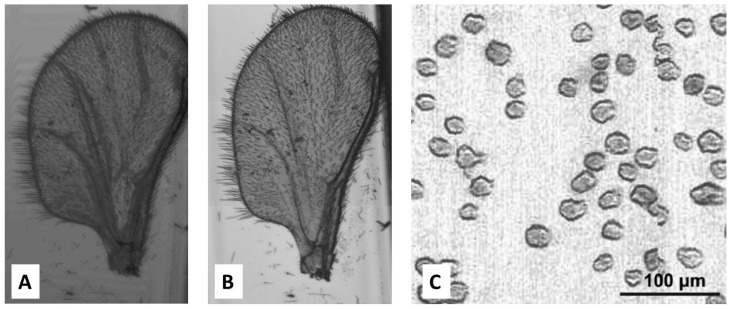
Comparison of images of a gnat wing obtained from (**A**) a CD-based system as a microscope and (**B**) a conventional bright field laboratory microscope. Adapted with permission of Taylor & Francis from [108]. (**C**) Demonstration of CD player as a diagnostic microscopy tool showing imaging of T2 cells (CD4+ and CD8-). Reproduced in part from [107] with permission of The Royal Society of Chemistry.

CD-based microfluidic systems have also been demonstrated to be effective microscopes [107,108]. Figure 8 gives some examples of images produced by CD-based systems utilized as microscopes, for a gnat wing [108], as well as for cell imaging [107].

For detection of low-concentration components in a sample, a solid phase extraction column can be embedded on a microfluidic disc for sample concentration. However, for certain assays, flow reciprocation, which uses stored pneumatic pressure to pump a sample back and forth over a detection chamber, is preferable. Noroozi *et al.* [49] used flow reciprocation to recycle a sample volume and pump it over an immunoassay array several times, maximizing the incubation of antigens and antibodies. The method was found to be more efficient than assays based on flow-through or passive diffusion methods [113]. Thermal cycling can also be implemented to carry out polymerase chain reaction (PCR) for DNA extraction [111].

Another intriguing use of the microfluidic disc is as a hypergravity simulator, albeit on the micro scale. Similar to the human centrifuges used by space programs for astronaut training, the disc can be used to simulate high g-forces on small biological organisms, such as *C. elegans* worms. Kim *et al.* [112] developed a microfluidic cultivation disc that subjected *C. elegans* to hypergravity environments as high as 100 *g* in order to study the stress response of organisms.

### 4.2. Innovative Centrifugal Approaches for Extreme Point-of-Care

In addition to the capabilities of centrifugal microfluidics to execute a variety of laboratory equipment functionality on a single system, a number of additional innovative approaches for result analysis, powering of systems and environmental control have been or are in the process of being explored.

#### 4.2.1. Sensing and Detection

Both optical and electrochemical sensing and detection techniques have been applied to centrifugal microfluidic systems. A brief overview of both approaches is summarized here, with clear advantages of low-cost and robust electrochemical techniques for extreme POC settings presented.

Optical read-out or detection technologies for centrifugal microfluidics have been reviewed in detail [114,115]. Absorbance measurements, based on changes in optical density, are amongst the most commonly used on the lab-on-a-disc platform. Grumann *et al.* used a total internal reflection (TIR) mirror-based system to increase the on-disc optical path length to make sensitive glucose measurements from human blood [116]. Similarly, Nwankire *et al.* used a 3D-printed portable spin stand with integrated absorbance measurements to implement a six-parameter liver assay panel from whole blood [117]. Absorbance is also widely used in environmental monitoring [118,119]. Colorimetric measurements methods have also been used for this application [120].

Fluorescence is amongst the most widely-used detection methods in biomedical diagnostics and has been demonstrated on-disc for applications, such as fluorescent immunoassays [121,122]. Similarly, the previously-described technique based on bead sedimentation [90,91] also used fluorescence as the detection method.

Other optical measurement techniques that have been used on the centrifugal platform include optical disc drive components [123], flow visualization using stroboscopically-coupled CCD cameras [124], color changes in paper strips [84] and Raman spectroscopy [125].

For extreme POC applications, bulky and expensive external instrumentation is not desirable, limiting some optical set-ups for these applications. Electrochemical detection methods, which are inexpensive, portable and have a low equipment footprint, are thus a favorable option for extreme POC settings. Additionally, fluorescent sensing on CD-based platforms often requires more expensive optical-grade polycarbonate discs, a parameter that does not affect electrochemical detection. The latest electrochemical sensors, such as amperometric sensors featuring redox amplification [126], have sensitivities and very low limits of detection (LODs) that are comparable to optical detection schemes, making them an attractive option for application in future POC systems. The advantages of electrochemical *versus* optical detection in extreme POC applications are summarized in Table 2.

**Table 2:** Comparison of optical *vs*. electrochemical detection techniques, compiled from information in a book series by Madou [108,127,128].

Parameter	Optical Sensors	Electrochemical Sensors
Instrument cost and size	Often expensive and bulky	Inexpensive and compact
Sensor cost	Fair	Low
Optically transparent substrate	Required	Not required
Selectivity	Good	Fair
Limit of detection (LOD)	Very good	Good and very good (using redox amplification
Response time	Long (up to tens of seconds)	Less than a second
Simplicity of the method	Often simple	Simple
Analysis of turbid solutions	Sometimes problematic	Not problematic
Electromagnetic interface	No	Yes
Resistance to radiation and corrosion	Yes	No
Cross-talk	No	Yes
Ambient light	Problematic	Not problematic
Response curve	Sigmoidal	Nernstian (potentiometric or linear)
Sensitivity enhancement	Complicated	Nernstian (potentiometric or linear)

**Figure 9:**
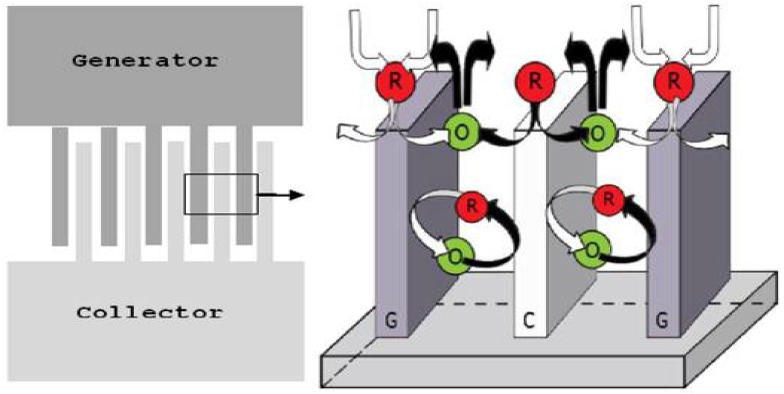
Three-dimensional carbon interdigitated electrode arrays (IDEAs) for redox amplification. For two adjacent electrodes in this configuration that are closely spaced and biased at different potentials, species will undergo oxidation at the electrode with a higher potential or the generator (G), and oxidized species will undergo reduction at the electrode with a lower potential or the collector (C). For G and C electrodes that are in close proximity, redox species can undergo redox cycling multiple times before they diffuse out into the bulk solution. Adapted with permission from [126]. Copyright 2014 American Chemical Society.

One of the most attractive qualities of electrochemical detection is the cost and size of the platform. Newly-developed electrochemical sensors, such as carbon sensors, can be made using inexpensive materials and fabrication methods to realize compact sensing devices. The external detection equipment can also be simplified: a combination of a low-cost miniaturized potentiostat [129] and inductive power transfer and wireless data transfer [58] is ideal for POC applications. For optical detection, miniaturization can be a challenge, as the optical path length and light intensity are reduced. In electrochemical detection, miniaturization enhances the sensitivity and LOD by reducing the capacitive current and, thereby, increasing the signal-to-noise ratio. In addition, electrochemical sensors have faster response times than optical sensors [108]. For example, for measuring dissolved oxygen, optical sensors have a typical response time of 40 s, while electrochemical sensors have a response time of 8–18 s [130]. The three-dimensional carbon microelectrodes developed by Kamath *et al.* [126] display, on the one hand, a wide stability window and low fabrication costs, and on the other hand, they further increase sensitivity by using a technique known as redox cycling, a recurring electrochemical reaction of a reversible redox coupled between two adjacent microelectrodes, as illustrated in Figure 9. This technique significantly amplifies the current generated in the electrochemical cell up to 40 times and allows these electrochemical sensors to operate at sensitivities comparable to optical detection. The combination of these highly selective and inexpensive electrochemical sensors, along with the general ease of use and simplicity of electrochemical detection, makes electrochemistry ideal for integration into POC devices.

**Figure 10:**
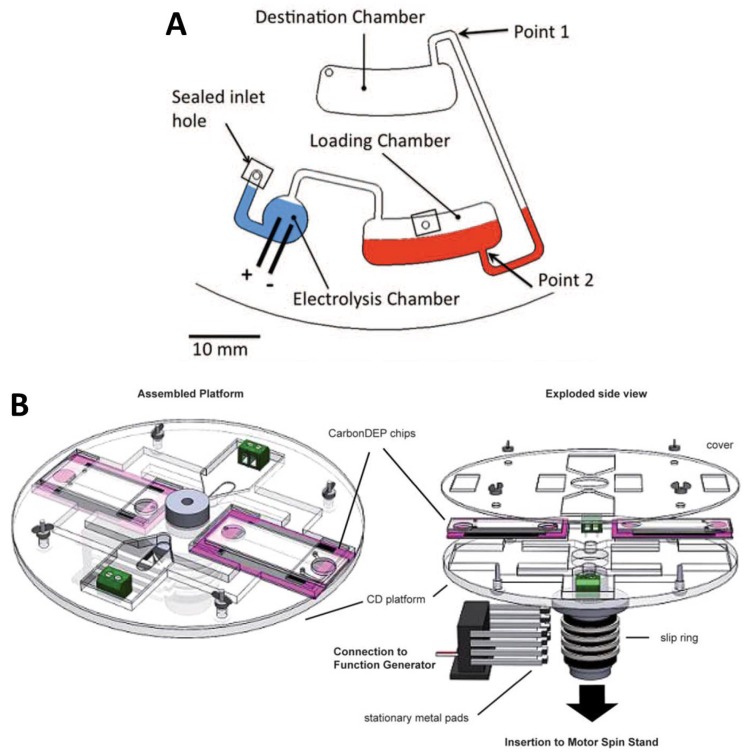
Electrochemistry implemented on CD-based systems. (**A**) Fluidic handling through electrolysis-generated pneumatic pressure [76]. Electrolyte and sample are loaded and localized at the bottom of their respective chambers as a result of spinning the disc. Electrical potential applied to the electrodes in the electrolysis chamber causes the sample to rise along the radial chamber to Point 1, completely transferring the sample to the destination chamber as long as electrical potential is applied. Reproduced in part by permission of The Electrochemical Society. (**B**) Carbon electrode dielectrophoresis (DEP) for trapping particles of interest. The set-up consists of a CD platform mechanically connected to a motor through a custom-made spin chuck and slip ring configuration for electrically connecting a function generator to the spinning disc. The CD houses an electrical circuit to interface the signal from the function generator to the DEP chips that are positioned in slots on the CD device. The chips contain DEP electrodes and microfluidic networks, providing a modular experimental set-up. Reproduced from [140] with permission of The Royal Society of Chemistry.

Electrochemical detection has already been successfully integrated onto the lab-on-a-disc platform using a slip ring-and-brush set-up [76] or a low-noise slip ring with liquid mercury [131]. Electrochemistry has been used in centrifugal microfluidic systems for glucose sensing [132], to detect proteins in bodily fluid [133], to perform rare cell detection [88], for pumping through electrolysis [76] and for flow monitoring [134]. Examples of electrochemistry implemented on CD-based systems are illustrated in Figure 10. Future schemes could integrate electrochemical sensors into total analysis discs for detection in common immunoassays, such as enzyme-linked immunosorbent assay (ELISA), and even as electrochemical DNA biosensors [135,136,137,138,139].

#### 4.2.2. Energy for Operation

Lab-on-a-disc set-ups can be designed in such a way that they encompass all components required for obtaining measurement results, including the necessary actuators, sensors and analytes. However, this capability entails high costs, which might be unacceptable in the context of extreme POC situations, which are remotely situated. Since the lab-on-a-disc equipment is integrated into one device, it contains specialized components, which can only be maintained and repaired by the manufacturer. The high cost of transporting a technician to remote settings to service such devices implies that this would only be a feasible solution if sufficiently many devices were deployed in a specific region. To address this issue, it is necessary to reduce the complexity of these lab-on-a-disc devices and to manufacture them with integrated self-test and calibration routines. In addition, these systems would be built in a modular way that allows maintenance to be performed by trained locals, with low-cost replacement modules readily available.

To date, the majority of lab-on-a-disc applications use only passive elements on the spinning disc, while all interactions with the disc are performed using stationary sensors and actuators. This presents challenges, such as bridging the gap between the instruments and the disc, as well as managing small duty cycles as a result of the rotation of the disc, which leads to expensive, highly sensitive and power-hungry device requirements. With new advances in wireless power and signal transfer, steps to overcome these limitations have been made, allowing for power and data connectivity to be integrated into centrifugal microfluidic systems [58]. Among other applications, this would enable the operation of electrochemical electrodes on a spinning disc.

In the context of remote POC applications, low-cost and maintenance-free continuous operation is of paramount importance, and the approach of incorporating power and a microcontroller onto the spinning disc assists in addressing these needs. The on-board logic, sensing and actuation capabilities allow for smaller, encapsulated and, hence, more reliable components to be included. The availability of a microcomputer also facilitates the implementation of test and calibration routines, which could aid local staff with minimal training to carry out repairs. In addition, the necessity for highly accurate speed control of the spinning motor would decrease, since the actual centrifugal forces on the disc could be measured directly, and the propagation of the fluidic interface could be used as a control trigger signal directly.

To reduce cost and personnel training efforts, the centrifugal microfluidic system could transfer and store data by utilizing standard technology, such as Bluetooth and Secure Digital (SD) card modules. These components typically have a combined power consumption of 200 mW. Control of the application could be achieved through an Arduino platform that operates at less than 190 mW. Power could be supplied to the application through close-range wireless power transmission methods, like the 5 W Qi-standard (Wireless Power Consortium), which can achieve an efficiency of greater than 80% through electromagnetic induction. Table 3 shows that, after taking into account the losses of the wireless power transmission and the other modules mentioned, there is still more than 3600 mW available for the particular application to be implemented on the centrifugal disc.

**Table 3:** Power management of an electronic disc for lab-on-a-disc applications.

Module	Power (mW)
Qi transmitter: Transmitted power	5000
On-disc power: Received power (80% efficiency)	4000
Arduino microcontroller consumption	190
Bluetooth and SD Card consumption	200
Energy available for application	3610

As a point of comparison, the energy of four fully-charged general-purpose AA batteries (1.5 V at 1000 m·Ah) supplying an Arduino microcontroller while sending data via Bluetooth from any application that consumes 2 W and, subsequently, storing the transmitted data on an SD card would last approximately only 2.5 h, which would be insufficient for running tests throughout the day in remote clinics.

The remaining 3.6 W enables the implementation of general applications. As an example, it would be expected that sensing based on current integrated electronic components would consume at most 100 mW. For applications where actuation is required, the example of implementing a PCR, which is commonly required for sample processing, can be considered. In order to perform heat cycling from 95 to 5 C in a small chamber of two cubic millimeters filled with 2 μL of water (4.18 kJ/(kg·K)), using for example the micro-Peltier element MPC-D403 (Micropelt, Freiburg, Germany) that consumes 1.4 W and transfers 500 mW as heat, 0.75 J needs to be provided. To avoid thermal drift of the Peltier element due to heat accumulation on its warm side, the Peltier could be mounted on a copper plane (top side) and connected through vias to a heat dissipator attached to a copper patch on the bottom side of the disc. The short cycle time of 1.5 s makes the implementation of on-disc PCR feasible using the power budget described.

**Figure 11:**
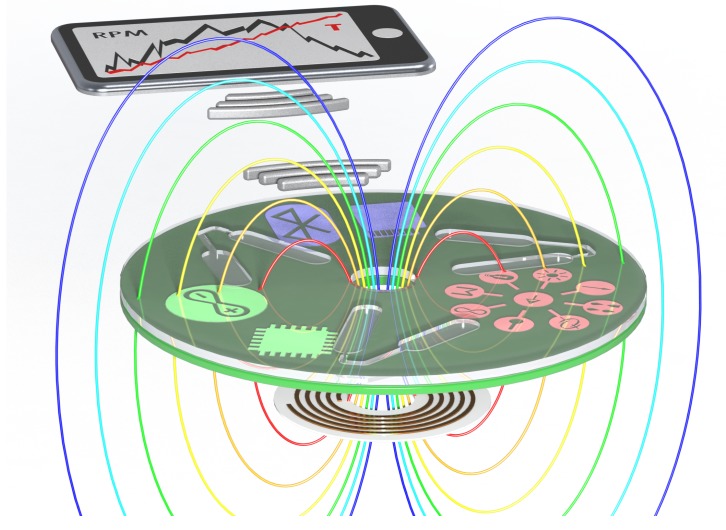
Concept design of smart power mechanisms for centrifugal microfluidics to allow for advanced functionality and connectivity on CD-based systems.

Figure 11 illustrates the overall concept of integrated smart mechanisms for power, data storage and connectivity for centrifugal microfluidic systems. A disposable disc can connect to an integrated system for powering of the application on the disc, with the result wirelessly transmitted to an external device (for example, a smart phone).

#### 4.2.3. Biomimetic Approaches for Environmental Control

While the goal of POC systems has been to create total analysis systems that can be used in almost any environment, to date, there has been very little focus on how to create fully-functioning systems that can operate in various temperature, humidity and power ranges. One potential solution to this issue is the use of biomaterials or biomimetic materials inspired by nature.

Silk cocoon membranes and spider silk have been a main staple in the textile industry and, more recently, in the biomedical industry [141]. Silk cocoons are specifically designed by nature to keep developing pupae intact despite seasonal temperature changes, heavy rains and gas exposure. The same properties that make it an ideal incubation environment in nature can be utilized to protect POC diagnostic devices from the harsh temperature, humidity [142] and other environmental changes present in extreme settings without the need for external power. In addition, recent studies have shown that the presence of humidity and saturated environments can be harnessed to generate electricity by a cocoon membrane [143], illustrating the potential of utilizing silk cocoon membranes and silk fibers for small power generation and storage applications in POC systems. While the cost of silk production remains high, new methods are being developed to artificially create silk, lowering the cost of silk from several dollars per kilogram to below one dollar per kilogram [144], which could be an affordable option for future development of environmental control in POC diagnostics. For future developments, silk cocoons could be used in microfluidic discs for long-term storage of dry reagents, keeping reagents protected from the elements until they are ready to be used, and even in enclosing disposables or other equipment and regulating the temperature and humidity of a microfluidic system.

### 4.3. Evolution of the CD Platform

The advances and innovations of lab-on-a-disc technologies have evolved over time since the introduction of centrifugal microfluidic systems in the 1960s [145]. Research-based centrifugal microfluidic platforms have been established at many universities and institutes around the world [146], including those in developing world settings, for example South Africa [36]. Centrifugal set-ups or spin stands are implemented in laboratory environments for testing, and these are used as a basis on which to develop prototypes and products that can be scaled up for mass manufacture.

#### 4.3.1. Four Generations of Spin Stands

**Figure 12:**
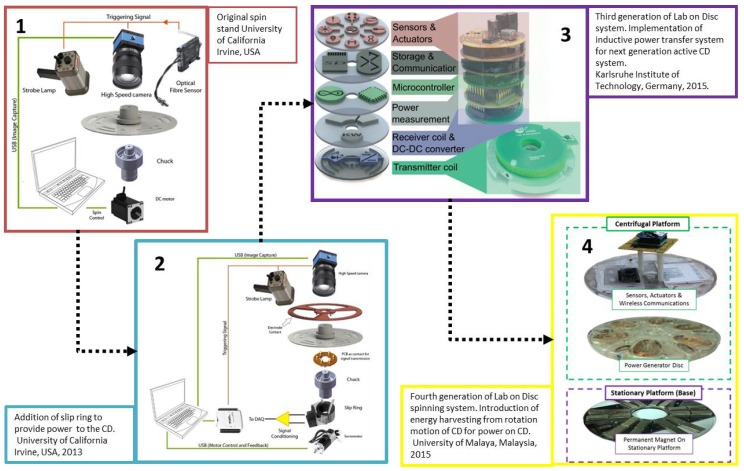
Progression of centrifugal microfluidic technology platforms towards stand-alone POC systems from different research groups. System 1 shows the original spin stand structure [38]. System 2 illustrates the addition of a slip ring to the spin stand set-up [140]. System 3 shows the implementation of an inductive power transfer system, reproduced in part from [58] with permission of the Royal Society of Chemistry. System 4 illustrates energy harvesting through the rotating motion of the disc [147].

Centrifugal system set-ups or spin stands have progressed over time in terms of functionality and sophistication. Figure 12 illustrates the progression of centrifugal microfluidic technology platforms towards stand-alone devices for extreme POC applications stemming from a number of different research and development institutions. Different types of spin stand set-ups each have advantages and disadvantages, as summarized in Table 4. Traditional set-ups utilizing slip rings are described (Image 2 of Figure 12), as well as systems utilizing inductive power transfer (as discussed in Section 4.2.2 and illustrated in Image 3 of Figure 12). In addition, CD-based systems implementing energy-harvesting techniques, recently presented by Joseph *et al.* [147], are also discussed.

**Table 4:** Comparison of different spin stand generations, highlighting the advantages and disadvantages of the slip ring set-up, wireless power transfer with Qi and energy harvesting. Plus (+) and minus (−) symbols indicate pros and cons, respectively, for the different categories listed on the left in the table, with neutral aspects indicated by +/−.

Parameter	Slip Ring	Wireless Power Transfer with Qi	Energy Harvesting
Wear	− contacts wear	+ no wear	+ no wear
Direct analog output	+ signals can be coupled in and out directly	− only digital signals can be sent to and from the disc	− only digital signals can be sent to and from the disc
Energy level	+ high voltages and currents can be sent to disc	+/− 5–20 W of power can be transferred (limited power)	+/− 100–500 mW of power can begenerated (relatively low power)
Constant energy	+ available energy does not depend on spinning frequency	+ available energy does not depend on spinning frequency	− induced power depends on the spinning frequency and is zero when stationary
Energy storage needed	+ no storage is needed since power is always available	+ no storage is needed since power is always available	− storage is needed, otherwise there is no power before spinning
Weight	+ the rotational part can be made to be light weight	+ the rotating disk is light weight	− the coils are heavy; hence, a high−torque motor is needed
Maintenance	+/− moderate maintenance	+ low maintenance	+ low maintenance
Price	+/− electrical brushes and contacts are mechanically complex	+ low cost, below EUR 50	+ low cost, below EUR 50
Interaction with surrounding	+ no effect on the surrounding	+/− RF might disturb some applications, but frequency can be adapted	− magnets on the lower disk inhibit magnetic sorting applications
Applicable to standard spin stands	− can only be integrated with a special spin stand head	+/− has only a few geometrical demands to enable integration	+/− has only a few geometrical demands to enable integration
Power source	− additional power source required	− additional power source required	+ no need for additional power source

#### 4.3.2. Scale-Up of CD-Based Microfluidic Systems

Requirements of microsystems for low-income POC were discussed at length by [148] and include the ability of these systems to utilize materials of a very low cost while maintaining robust functionality to cope with harsh handling, storage and transportation conditions in temperature ranges of 4–40 °C. All fluidic functionality should be automated, and detection should be low-cost, portable and part of a self-contained system. As discussed in the previous sections, CD-based microfluidic systems, combined with recent advances and innovations applied to these systems, have the potential to conform to these requirements.

Advances and challenges of centrifugal systems, with emphasis on commercialization aspects, have been discussed extensively [149]. System integration of microsystem technologies was investigated by Sin *et al.* and shows many advantages of centrifugal microfluidic systems for system integration, also when compared to other microfluidic technologies [39]. Centrifugal microfluidic systems perform well in terms of throughput (number of samples analyzed in a single assay), multiplexity (number of parameters tested for each sample) and diversity (variety of fluidic operations), as discussed by Sin *et al.*, giving centrifugal microfluidics an overall high performance rating [39]. Centrifugal microfluidic systems are also favorably positioned for mass production and commercial roll-out, as they can make use of existing equipment (laboratory centrifuges, *etc.*), meaning that the instrumentation required is accessible and widely accepted [150], making for more efficient fall-in with existing technologies and mindsets in the healthcare industry.

Massive integration and parallelization of microfluidic operations is a prime objective for POC devices where several tests or a range of samples could be performed on a single platform. Miniaturization using microfabrication and functional materials is key towards enabling valving technologies that will eventually perform a multitude of laboratory unit operations on a single lab-on-a-disc unit. Large-scale integration on more traditional platforms has previously been demonstrated [151]. Using multilayer soft lithography, Quake *et al.* showed the advantages of multiplexing with a combination of several pneumatically-controlled PDMS microvalves.

On centrifugal microfluidic systems, there has been significant progress on the microfabrication technologies that can enable large-scale operations on a single unit. Mark *et al.* have demonstrated prototyping of microfluidic cartridges made out of polymer films using microthermoforming by soft lithography for aliquoting of liquids with precise metering to the scale of individual 10-μL samples [61]. The Gyros Bioaffy platform has demonstrated liquid volume handling operations as low as 15 nL and a total of 104 immunoassays on a single disc [152]. The platform is based on centrifugal force-based delivery of reagents controlled by hydrophobic barriers to a bead-based sandwich immunoassay column, thus requiring specialized surface treatment. Kinahan *et al.* have demonstrated a networked relay system for valve triggering for multiple valves in a series, paving the way towards more complex process handling for multi-step assays on the centrifugal platform [69]. The technology is based on centrifugo-pneumatic valving that can also be operated in a logical gate structure allowing for further complex microfluidic operations, like parallelization and serial cascades. Given the increasing scope of application of large-scale integration for POC devices, the development of such platforms will be one of the defining challenges in centrifugal microfluidics.

#### 4.3.3. Examples of Existing Centrifugal Systems for Point-of-Care

A number of research- and development-based centrifugal microfluidic systems have showcased highly functional systems towards POC diagnostic applications, including a fully-integrated system for analysis of biochemistry and immunoassays using whole blood [54]. Recently, an automated bacterial pathogen detection system using PCR and DNA extraction on a disc was presented [153].

**Figure 13:**
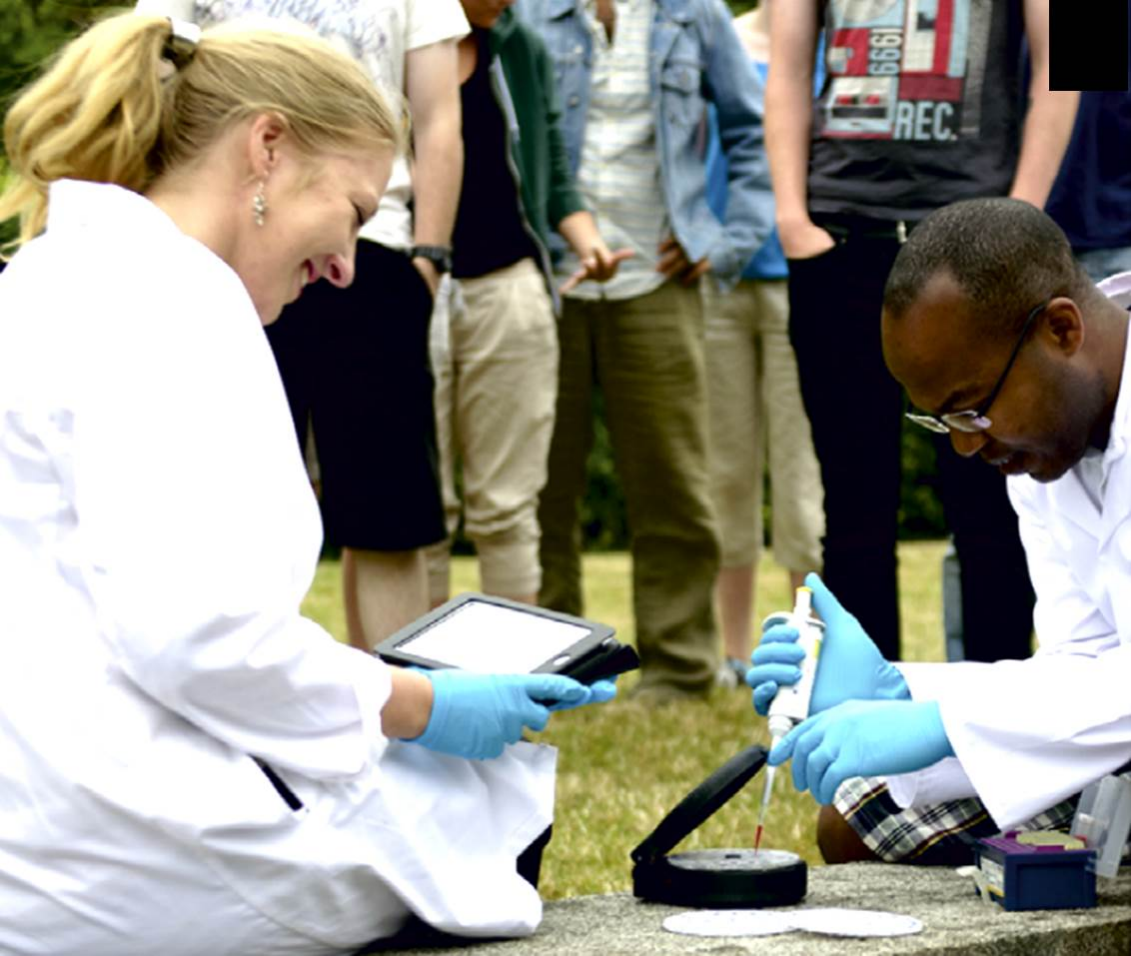
Testing of portable centrifugal microfluidic system for POC liver function testing in Nigeria. Reprinted from [117] with permission from Elsevier.

Nwankire *et al.* describe a portable centrifugal system for liver function testing that was deployed successfully in a lab environment in Nigeria, showing the potential of centrifugal microfluidic systems to perform well in under-resourced clinical settings [117] (Figure 13). Similarly, towards POC HIV diagnostics in Sub-Saharan Africa, Glynn *et al.* demonstrated isolation and semi-quantitative enumeration of CD4+ cells using centrifugo-magnetophoretic processing [154]. The company Abaxis started its centrifugal-based blood chemistry analysis system development more than 20 years ago [155] and is one of the few commercial centrifugal-based systems available today. The Abaxis Picollo Xpress blood chemistry analyzer has recently been used to support testing of Ebola [156,157]. Sharma *et al.* also explores this system as a POC diagnostic suited to low-resourced settings [99].

Other commercial centrifugal systems for diagnostics include the Samsung LABGEO IB10 [158], which supports various tests. GenePOC [159] is in the process of developing products, and POC Medical is working on a product for breast cancer detection [160]. In addition, Biosurfit SA from Lisbon, Portugal, just entered the market with a C-reactive protein (CRP) assay [161]. A comprehensive list of centrifugal microfluidic platforms that are currently commercially available, or nearing commercialization, is given by Strohmeier *et al.* [37]. The potential for centrifugal microfluidic systems to provide POC diagnostic solutions for extreme environments and specific life-threatening diseases has been demonstrated [99].

## 5. Ideal Panel of Tests for Extreme Point-of-Care

Centrifugal microfluidic systems have seen some success as POC solutions, and their capacity to be applied to extreme POC settings has been highlighted in this work. Lab-on-a-disc systems that form part of an integrated primary care solution have the potential to make an impact in under-resourced settings, such as rural clinics in India and South Africa.

The benefits of having a comprehensive primary care solution that leverages advanced technology are manifold. In addition to diagnosing and treating infectious diseases, the effective management of non-communicable diseases (NCDs), such as diabetes, hypertension, arthritis, *etc.* is also achievable. This has a far-reaching impact on DALYs saved, improvement in major health indices (e.g., life expectancy), societal productivity, reduced expenses through judicious use of drugs (due to better diagnosis) and, most importantly, reduction of healthcare-induced poverty.

To explore this potential impact, the magnitude of mortality of children under five years in India can be considered. Three causes accounted for approximately 78% of all neonatal deaths in India: (1) prematurity and low birth weight (32.7%), (2) neonatal infections (26.7%) and (3) birth asphyxia and birth trauma (18.8%). Two causes accounted for 50% of all deaths at ages 1–59 months: (1) pneumonia (27.6%) and (2) diarrheal diseases (22.4%) [162].

Prematurity and low birth weight issues can be addressed by correction of maternal anemia, while neonatal infections, birth asphyxia and trauma can be reduced through health education regarding institutional delivery. Pneumonia and diarrheal diseases can be diagnosed and treated at primary care centers equipped with appropriate POC diagnostics, and thus, primary care together with POC diagnostic technologies will have a multiplier effect on addressing child mortality issues and, thus, the population and society as a whole.

Lab-on-a-disc technologies could be applied to implement technology for realizing effective primary care in extreme or under-resourced settings. Table 5 illustrates that CD-based or lab-on-a-disc technologies are poised to significantly contribute to realizing the majority of critical tests required in extreme POC settings. The critical panel was compiled by one of the authors of this paper (Dr. Satadal Saha, a medical doctor, and founder of a number of clinics in rural India), along with inputs from his colleagues. In addition, critical tests for rural clinics in Africa using prevalent diseases as guidelines were also included.

The tests on the critical panel that have not been implemented on lab-on-a-disc platforms can utilize existing centrifugal microfluidic operations, such as bacterial detection and cell separation, towards the realization of these tests (see, for example, [37]). Where tests on the critical panel have not yet been addressed by centrifugal microfluidic technologies, future work on these technologies should focus on these areas for contributions to be made for extreme POC clinic settings.

The need for a fever panel of tests has also recently been highlighted, stemming from a high occurrence of respiratory infections across rural India [171]. A panel of tests has been formulated by Dr. Satadal Saha and his colleagues to allow for the distinction to be made between the different causes of the infection and, thus, fever. Such a panel would be applicable to patients that present with a fever that cannot immediately be ascribed to a specific cause, e.g., an abscess or an obvious infection, such as a urinary tract infection or tonsillitis. A panel of tests is thus required to diagnose or allow for differentiation between the following fever-causing diseases: malaria, dengue, typhoid, fever of viral origin TB, H1N1.

Tests for malaria and TB also form part of the critical panel, again showing the importance of developing effective POC tests for diagnosing these diseases and guiding the focus of POC technology development, particularly on centrifugal microfluidic platforms, moving forward.

**Table 5:** Critical panel of tests required for effective POC diagnostics in extreme settings and corresponding existing CD-based versions. Additional tests for the critical panel that still require CD-based implementations included malaria, thyroid function test (T3, T4, TSH) and typhoid fever test.

Critical Test for Under-Resourced POC	Lab-on-Disc Implementation?	Methods and References
Complete blood count	Yes	Cell capture and counting [94]. Blood fractionation, density gradient tests [87,88,89]. Hemoglobin [163] and Hematocrit [164]. From these tests, all remaining complete blood count values can be calculated.
Blood group (ABO and rhesus)	Yes	Agglutination of cells [165].
Blood sugar (F and PP ), urea, creatinine, uric acid	Yes	Colorimetric glucose assay [116]. Uric acid, glucose and lactate tests using whole blood and electrochemistry [166]. Urine analysis [167].
Serum sodium, potassium	Yes	Plasma separation and automated assay [155]. Commercially available: Abaxis Picollo Xpress.
Liver function test: bilirubin, liver enzymes (SGOT, SGPT, SAKP)	Yes	Liver function on a disc [117].
HbA1C (diabetes)	Yes	Commercially available: Roche Cobas b 101.
HbsAG (hepatitis B)	Yes	Integrated ELISA for detecting antigens and antibodies of hepatitis B virus, HBsAg and anti-HBs in parallel using whole blood [168].
HIV	Yes	CD4+ cell counts [154,169].
TB	In progress, some existing components.	Bacterial pathogen detection [170].
Urine-routine and microscopic, culture	In progress, some existing components.	Individual cell capture: counting can be performed using microscopy [94]. ELISA from cell culture [121].

## 6. Summary and Conclusions

The increasing burden of disease, particularly in under-resourced settings, such as India and Africa, demands effective diagnostic capabilities at the POC. However, the challenges faced in these under-resourced or extreme settings are vast, highlighting the need for and drive towards the development of dedicated technologies that directly address these requirements, as solutions for primary care in remote settings.

Microfluidic systems are generally well suited for POC solutions, as they utilize small volumes of sample and reagent in compact, disposable devices. Centrifugal microfluidic platforms are particularly beneficial in terms of POC applications due to their simplicity and the effectiveness of the instrumentation for fluidic control. This work discussed the various challenges in rural settings that require dedicated solutions for application to extreme POC scenarios and highlighted the potential of centrifugal or CD-based diagnostics to be applied here. However, a number of challenges still need to be overcome to make these systems truly viable in these settings.

Important technical challenges to be addressed include the use of equipment in environments with large fluctuations in temperature and humidity. Additionally, criteria to be addressed include performing diagnostic tests within required time limits, as well as the need to evaluate how well these tests can be implemented by local and possibly untrained staff at the rural clinics.

Centrifugal microfluidic systems have evolved substantially in the recent decade, resulting in a platform that has the potential to address many of the challenges faced in under-resourced clinic settings. A stand-alone lab-on-a-disc platform could be deployed to rural clinics in India or Africa to facilitate POC diagnosis in remote settings for routine microscopy and other sample preparation and analysis required for quick diagnosis or screening tests. Towards more robust diagnostic systems, innovative approaches on lab-on-a-disc platforms for POC, such as sensing and detection, smart power sources and environmental control, have been presented in this work. A foundation for effective extreme POC solutions is realized by joining novel solutions with existing centrifugal systems for POC and by the ability of these systems to be scaled up.

By applying centrifugal microfluidic advances to critical test panels formulated by medical experts in the field, it is evident that centrifugal microfluidics are poised to contribute significantly to making such test panels a reality in extreme clinic settings, potentially through integrated, stand-alone systems that are affordable and accessible. Leveraging CD-based microfluidic capabilities in this way to enable comprehensive primary care to be implemented could greatly impact extreme POC and the lives of those living in such under-resourced communities.

The focus required to move centrifugal fluidic development forward should be on environmental control and effective powering of devices, which are critically important aspects in extreme settings. Implementation of diagnostic tests that have not yet been covered by existing CD-based technology should also be pursued to provide a complete critical test panel.

This work provides a first attempt at understanding the limitations of extreme POC settings and a first step towards formulating solutions. Working together with medical experts in the field assists in formulating comprehensive requirements, enabling an effective solution to be mapped and developed. In parallel and at an early stage of development, it is important to deploy CD-based systems in extreme POC environments and to assess their performance and failure conditions, either as a result of human error, environmental aspects or technical functionality. These issues should then be addressed by re-working the design and implementation of the CD-based systems, improving the success of these systems in extreme settings. Although a number of practical challenges still need to be solved, recent advances made in CD-based microfluidic technologies show promise as feasible solutions for extreme POC (EPOC) applications.
